# Supplementary material for: APE1 distinguishes DNA substrates in exonucleolytic cleavage by induced space-filling
Source: Nat Commun. 2021 Jan 27;12:601. doi: 10.1038/s41467-020-20853-2 (PMC7841161; doi:10.1038/s41467-020-20853-2)
Supplement: Supplementary file 1 — Supplementary Information [file 41467_2020_20853_MOESM1_ESM.pdf]

Supplementary information

for

APE1 Distinguishes DNA Substrates in Exonucleolytic Cleavage by Induced Space Filling

Tung-Chang Liu[1,2], Chun-Ting Lin[3] , Kai-Cheng Chang[2], Kai-Wei Guo[2], Shuying Wang[4,5,6,7], Jhih-Wei Chu[1,2,8,9] and Yu-Yuan Hsiao[1,2,3,8,9,10*]

[1] Institute of Molecular Medicine and Bioengineering, National Chiao Tung University, Hsinchu 30068, Taiwan

[2] Department of Biological Science and Technology, National Chiao Tung University, Hsinchu, Taiwan 30068, ROC.

[3] Master's and Doctoral Degree Program for Science and Technology of Accelerator Light Sources, National Chiao Tung University, Hsinchu 30068, Taiwan

[4] Department of Microbiology and Immunology, College of Medicine, National Cheng Kung University, Tainan, Taiwan

[5] Center of Infectious Disease and Signaling Research, National Cheng Kung University, Tainan, Taiwan.

[6] Institute of Basic Medical Sciences, College of Medicine, National Cheng Kung University, Tainan, Taiwan

[7] Department of Biotechnology and Bioindustry Sciences, College of Bioscience and Biotechnology, National Cheng Kung University, Tainan, Taiwan

[8] Institute of Bioinformatics and Systems Biology, National Chiao Tung University, Hsinchu, 30068, Taiwan

[9] Center For Intelligent Drug Systems and Smart Bio-devices (IDS^{2}B), National Chiao Tung University, Hsinchu, Taiwan

[10] Drug Development and Value Creation Research Center, Center for Cancer Research, Kaohsiung Medical University, Kaohsiung, Taiwan

Supplementary Tables: 1-3

Supplementary Figures: 1-12

Supplementary Table 1. Substrates for biochemical studies

Substrate	Sequence
ssDNA 11 nt	5′- AATCTTACAAA -3′
ssDNA 20 nt	5′- ACTGGACAAATACTCCGAGG -3′
dsDNA 20 nt	5′- ACTGGACAAATACTCCGAGG -3′<br>3′- TGACCTGTTTATGAGGCTCC -5′
dsDNA 20 mer with damaged DNA bases at the 11th nucleotide	5′- ACTGGACAAATXCTCCGAGG -3′<br>3′- TGACCTGTTTATGAGGCTCC -5′<br>X: tetrahydrofuran (abasic site analog), 8-oxoguanine (8-oxoG), or hypoxanthine (deoxyinosine)
dsDNA 20 mer with damaged DNA bases at 3′-end	5′- ACTGGACAAATACTCCGAGX -3′<br>3′- TGACCTGTTTATGAGGCTCC -5′<br>X: tetrahydrofuran (abasic site analog), 8-oxoguanine (8-oxoG)
dsDNA 20 mer with Biotin at 3′-end	5′- ACTGGACAAATICTCCGAGG B -3′<br>3′- TGACCTGTTTATGAGGCTCC -5′<br>I : hypoxanthine (deoxyinosine) ; B : biotin
dsDNA 20 mer with 1 nt mismatched	5′- ACTGGACAAATACTCCGAGG -3′<br>3′- TGACCTGTTTATGAGGCTCA -5′
dsDNA 20 mer with 2 nt mismatched	5′- ACTGGACAAATACTCCGAGG -3′<br>3′- TGACCTGTTTATGAGGCTAA -5′
Recessed dsDNA 20 mer with 5 nt 5′-overhang	5′- ACTGGACAAATACTCCGAGG -3′<br>3′- TGACCTGTTTATGAGGCTCCCACTA -5′
1 nt mismatched recessed dsDNA 20 mer with 5 nt 5′-overhang	5′- ACTGGACAAATACTCCGAGG -3′<br>3′- TGACCTGTTTATGAGGCTCACACTA -5′
Recessed dsDNA 20 mer with 20 nt 5′-overhang	5′- ACTGGACAAATACTCCGAGG -3′<br>3′- TGACCTGTTTATGAGGCTCCCACTAATCACTTCGCACGAG -5′
1 nt mismatched recessed dsDNA 20 mer with 20 nt 5′-overhang	5′- ACTGGACAAATACTCCGAGG -3′<br>3′- TGACCTGTTTATGAGGCTCACACTAATCACTTCGCACGAG -5′
2 nt mismatched recessed dsDNA 20 mer with 20 nt 5′-overhang	5′- ACTGGACAAATACTCCGAGG -3′<br>3′- TGACCTGTTTATGAGGCTAACACTAATCACTTCGCACGAG -5′
1-nt-gapped dsDNA 40 mer with 5′-OH (phosphoryl groups at 5′ margin)	5′- ACTGGACAAATACTCCGAGG HO-TGATTAGTGAAGCGTGCTC -3′<br>3′- TGACCTGTTTATGAGGCTCCC ACTAATCACTTCGCACGAG -5′
1-nt-gapped dsDNA 40 mer with 5′-phosphate (hydroxyl groups at 5′ margin)	5′- ACTGGACAAATACTCCGAGG PTGATTAGTGAAGCGTGCTC -3′<br>3′- TGACCTGTTTATGAGGCTCCC ACTAATCACTTCGCACGAG -5′
1-nt-gapped dsDNA 40 mer with 1 nt mismatch	5′- ACTGGACAAATACTCCGAGG PTGATTAGTGAAGCGTGCTC -3′<br>3′- TGACCTGTTTATGAGGCTCAC ACTAATCACTTCGCACGAG -5′
1-nt-gapped dsDNA 40 mer with 2 nt mismatch	5′- ACTGGACAAATACTCCGAGG PTGATTAGTGAAGCGTGCTC -3′<br>3′- TGACCTGTTTATGAGGCTAAC ACTAATCACTTCGCACGAG -5′
Nicked dsDNA 40 mer with 5′-OH (hydroxyl groups at 5′ margin)	5′- ACTGGACAAATACTCCGAGG ^HO-GTGATTAGTGAAGCGTGCTC -3′<br>3′- TGACCTGTTTATGAGGCTCC CACTAATCACTTCGCACGAG -5′
Nicked dsDNA 40 mer with 5′-phosphate (phosphoryl groups at 5′ margin)	5′- ACTGGACAAATACTCCGAGG ^PGTGATTAGTGAAGCGTGCTC -3′<br>3′- TGACCTGTTTATGAGGCTCC CACTAATCACTTCGCACGAG -5′
1 nt mismatch nicked dsDNA 40 mer with 5′-OH (hydroxyl group at 5′ margin)	5′- ACTGGACAAATACTCCGAGG ^HO-GTGATTAGTGAAGCGTGCTC -3′<br>3′- TGACCTGTTTATGAGGCTCA CACTAATCACTTCGCACGAG -5′
1 nt mismatch nicked dsDNA 40 mer with 5′-phosphate (phosphate at 5′ margin)	5′- ACTGGACAAATACTCCGAGG ^PGTGATTAGTGAAGCGTGCTC -3′<br>3′- TGACCTGTTTATGAGGCTCA CACTAATCACTTCGCACGAG -5′

*The underline regions are the paired region of DNA substrates.
* ^: nick site, P: phosphoryl group, HO: hydroxyl group

Supplementary Table 2. Crystallization conditions of mAPE1-dsDNA product complexes

**mAPE1-blunt-ended-dsDNA product complex**		
Protein：wild-type mAPE1<br>Input DNA[a]：5′-CGTAATACG-3′<br>Time for growth：3 to 5 weeks<br>Condition for crystallization :<br>0.1M Bis-Tris pH 6.5, 25 % v/v Polyethylene glycol 300	Input DNA	DNA in the structure [b]
	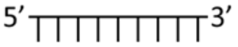	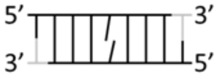
**mAPE1-recessed-dsDNA product complex**		
Protein：mAPE1Δ30<br>Input DNA[a]：5′-GCGTAATAC-3′<br>Time for growth：3 to 4 days<br>Condition for crystallization :<br>0.1M Lithium sulfate monohydrate, 0.1M Na citrate tribasic dihydrate pH 5.5, 20 % w/v Polyethylene glycol 1,000	Input DNA	DNA in the structure
	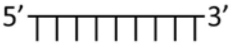	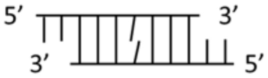

[a] The underline regions are paired regions of DNA substrates.
[b] DNA regions displayed in gray are the regions degraded by mAPE1

Supplementary Table 3. Crystallographic data and refinement statistics

**Structure**	**mAPE1-blunt-ended-dsDNA product complex**	**mAPE1-recessed-dsDNA product complex**
**PDB ID**	7CD5	7CD6
**Data collection**		
Space group	$P6_522$	$P6_322$
Cell dimensions		
a, *b*, *c* (Å)	107.34, 107.34, 230.68	124.65, 124.65, 138.47
α, β, γ (°)	90.0, 90.0, 120.0	90.0, 90.0, 120.0
Resolution (Å)	30.0-2.7 (2.8-2.7)	30.0-2.7 (2.8-2.7)
R_{sym} or R_{merge}	5.4 (39.5)	11.2 (45.9)
$I / \sigma I$	36.7 (2.9)	24.0 (2.5)
Completeness (%)	99.9 (99.6)	99.9 (99.6)
Redundancy	17.7 (13.5)	17.0 (8.9)
**Refinement statistics**		
Resolution (Å)	29.9-2.7	29.9-2.7
No. reflections	22276	17914
R_{work} / R_{free}	19.0/21.7	17.8/22.0
**B-factors (Å^2)**		
All	65.0	57.0
Protein	64.68	56.42
DNA	72.47	73.18
Water	-	46.57
R.m.s. deviations		
Bond lengths (Å)	0.005	0.006
Bond angles (°)	0.781	0.842
Ramachandran blot statistics (%)		
Favored region	97.83	95.64
Allowed region	2.17	4.36
Outlier region	0	0

[a] Each structure was obtained from single crystal
[b] Values in the parentheses are for highest-resolution shell

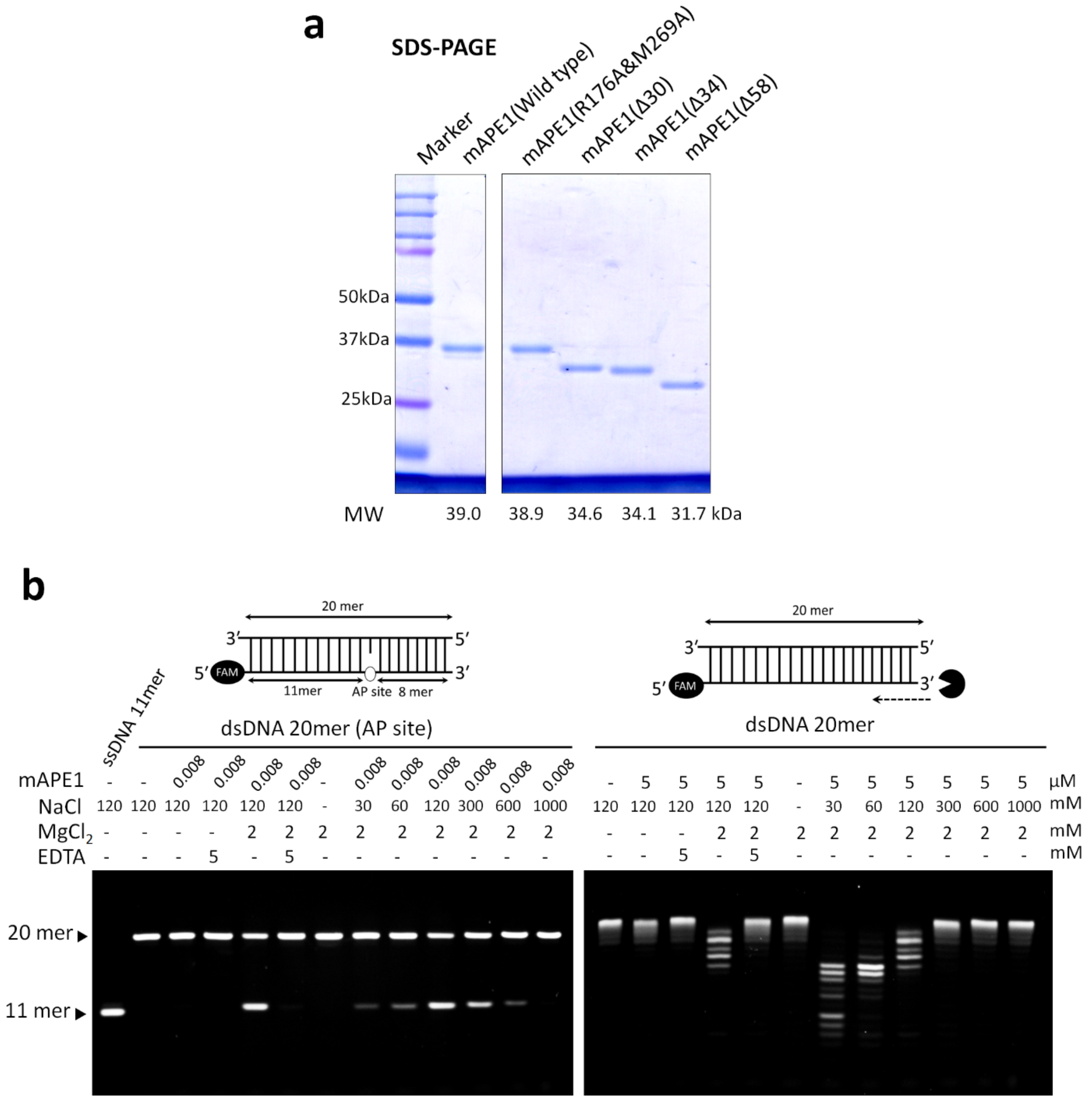

Supplementary Fig. 1: The basic biochemical properties of mAPE1. a The recombinant full-length, truncated and mutated mAPE1 were purified and protein purity was analyzed by SDS-PAGE. **b** The optimal conditions for the endo- and exonuclease activity assays of mAPE1. The optimal conditions for endo- and exonuclease activity are different. Under 120 mM NaCl and 2 mM $MgCl_2$, endonuclease activity is the highest. Exonuclease activity of mAPE1 on the other hand is higher under low NaCl concentration (30 mM) in the presence of 2 mM $MgCl_2$. **a-b** Source data are provided as a Source Data file.

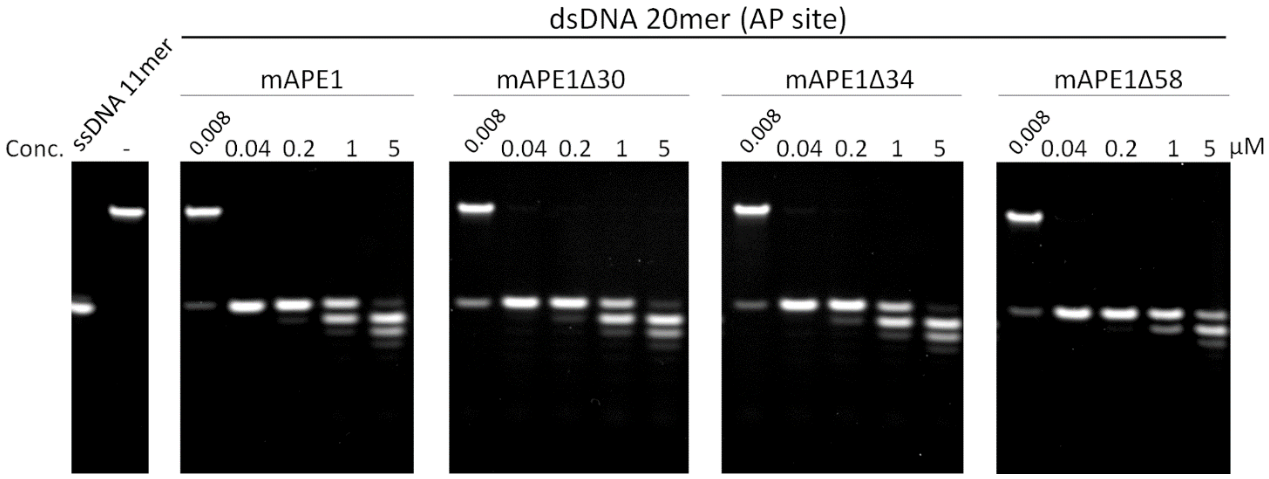

Supplementary Fig. 2: The endonuclease activity of full-length and truncated mAPE1. Various mAPE1 were incubated with dsDNA containing an AP site to measure the endonuclease activity of full-length and truncated mAPE1. The endonuclease activity of full-length and truncated mAPE1 displayed similar levels of activity on digesting AP site-containing dsDNA. mAPE1△58 showed lower exonuclease activity after the endonucleolytic digestion. Source data are provided as a Source Data file.

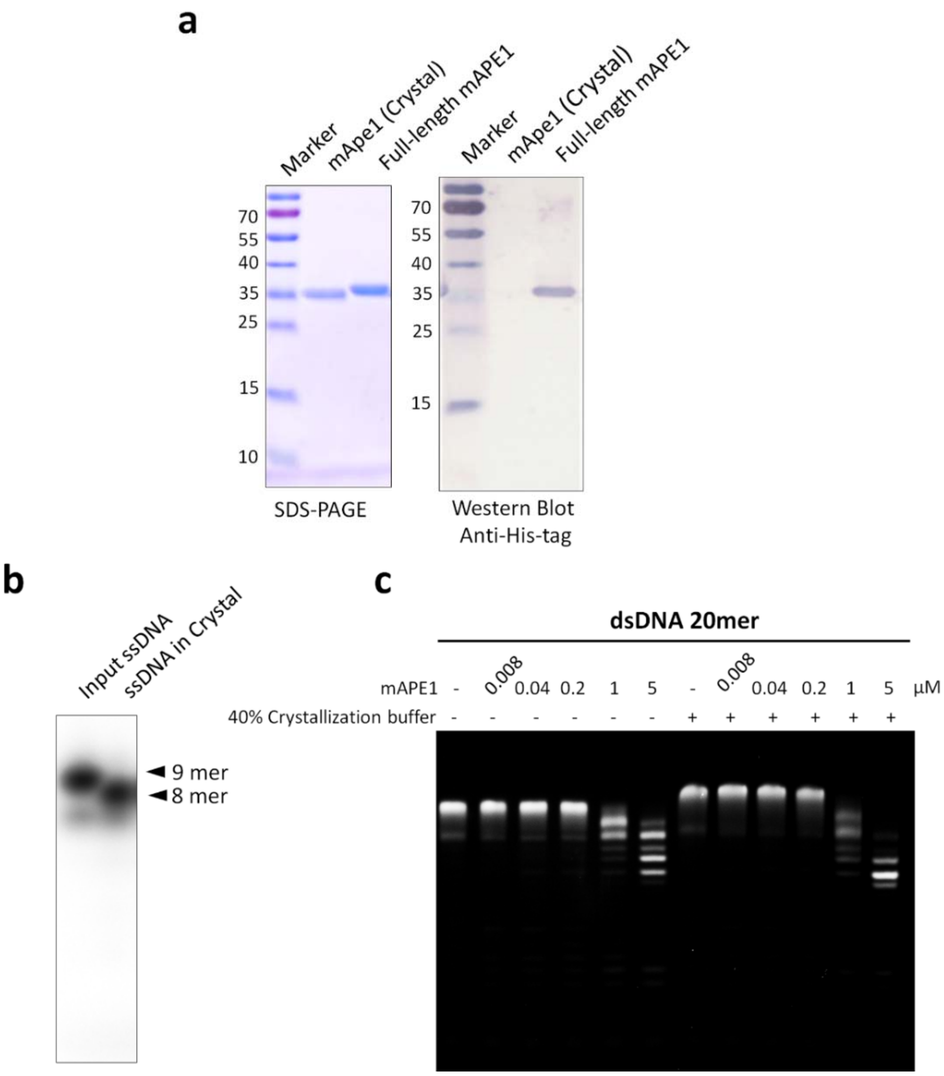

Supplementary Fig. 3: Gel-based analyses of the mAPE1 and DNA in the crystal. a SDS-PAGE and western blot analyses of the mAPE1 protein in the crystal of mAPE1-blunt-ended-dsDNA product complex. The molecular weight of mAPE1 in the crystal is lower than that of the full-length mAPE1. The crystallized mAPE1 also could not be detected by anti-His tag antibody in western blot experiments. Both evidence indicated the N-terminal His tagged mAPE1 was degraded from the N-terminal during the crystallization process. **b** Denaturing urea polyacrylamide gel electrophoresis of the isotope-labeled input DNA and the DNA in crystal. The last nucleotide at the 3′-end of DNA was removed during the crystallization process. **c** Exonuclease activity of mAPE1 on dsDNA degradation under crystallization buffer condition. The result shows that the activity of mAPE1 is slightly enhanced in the presence of crystallization buffer condition. **a-c** Source data are provided as a Source Data file.

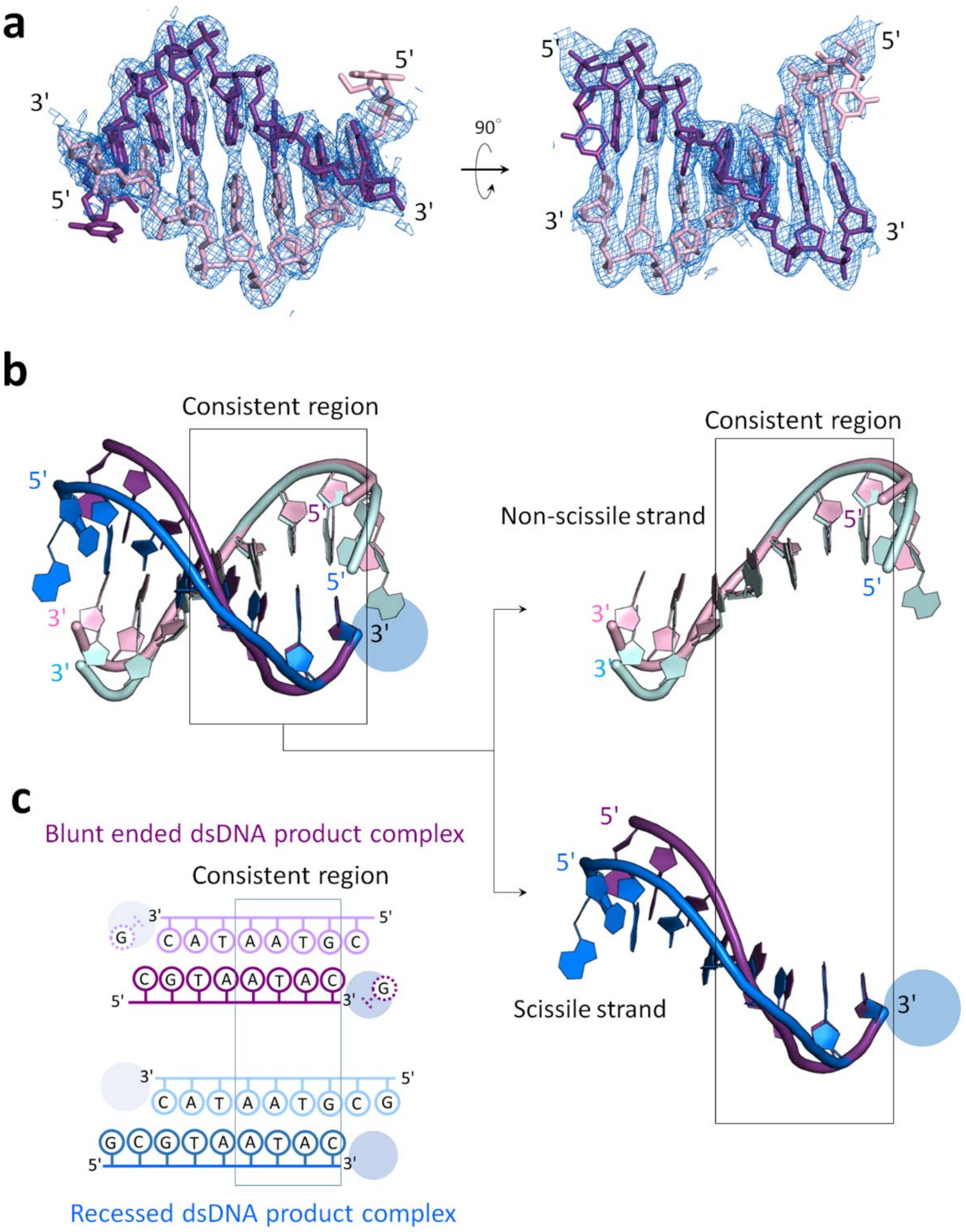

Supplementary Fig. 4: The structural analyses of dsDNA in the structures of mAPE1-dsDNA product complexes. a The omitted electron density map (Fo − Fc, 2.0 σ) of dsDNA in the mAPE1-blunt-ended-dsDNA product complex. **b-c** Structural alignment of blunt-ended and recessed dsDNA in our two mAPE1-dsDNA product complexes. The structure of four base pairs extending from the active center is highly consistent in the two structures. The 5′-overhang regions and the base pairs away from the active site are not superposed well between these two product complex structures.

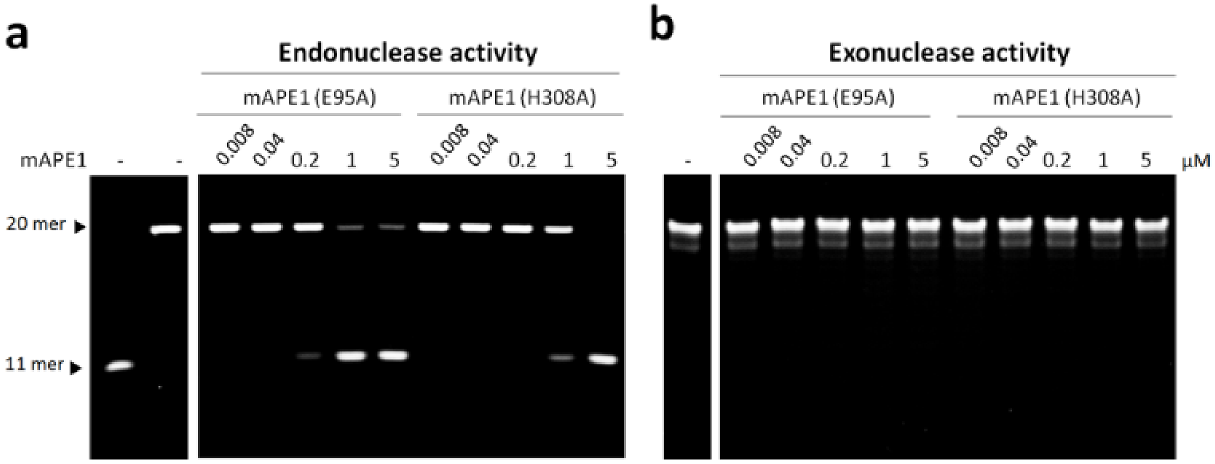

Supplementary Fig. 5: The endo- and exonuclease activity assays for mAPE1 active site mutants. **a-b** The endo- and exonuclease activity of active site mutants, E95A and H308A, were measured. In wild-type mAPE1, the patterns for endo- and exonucleolytic digestion can be observed at the enzyme concentration of 0.04 and 1 μM, respectively (Fig. 2). The patterns of endonucleolytic digestion of E95A and H308A are observed at the enzyme concentration of 0.2 and 1 μM, respectively. The exonuclease activity of the two mutants cannot be observed even the enzyme concentration reaches to 5 μM. Both the endo- and exonuclease activity of E95A and H308A mutants are lower than that of wild-type mAPE1. **a-b** Source data are provided as a Source Data file.

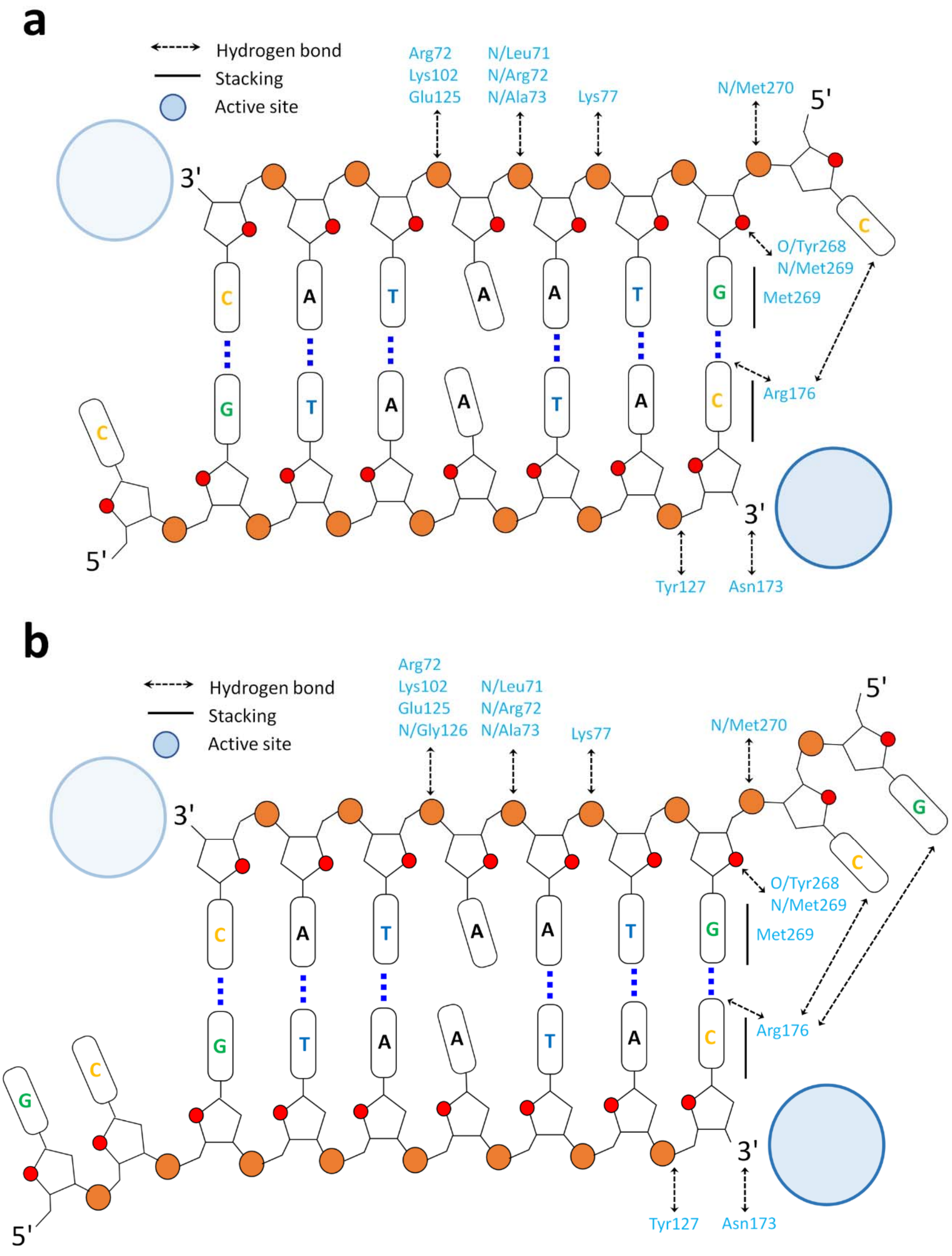

Supplementary Fig. 6: Schematic of the interactions between mAPE1 and structural DNAs. a For the structure of the mAPE1-blunt-ended-dsDNA product complex. **b** For the structure of the mAPE1-recessed-dsDNA product complex.

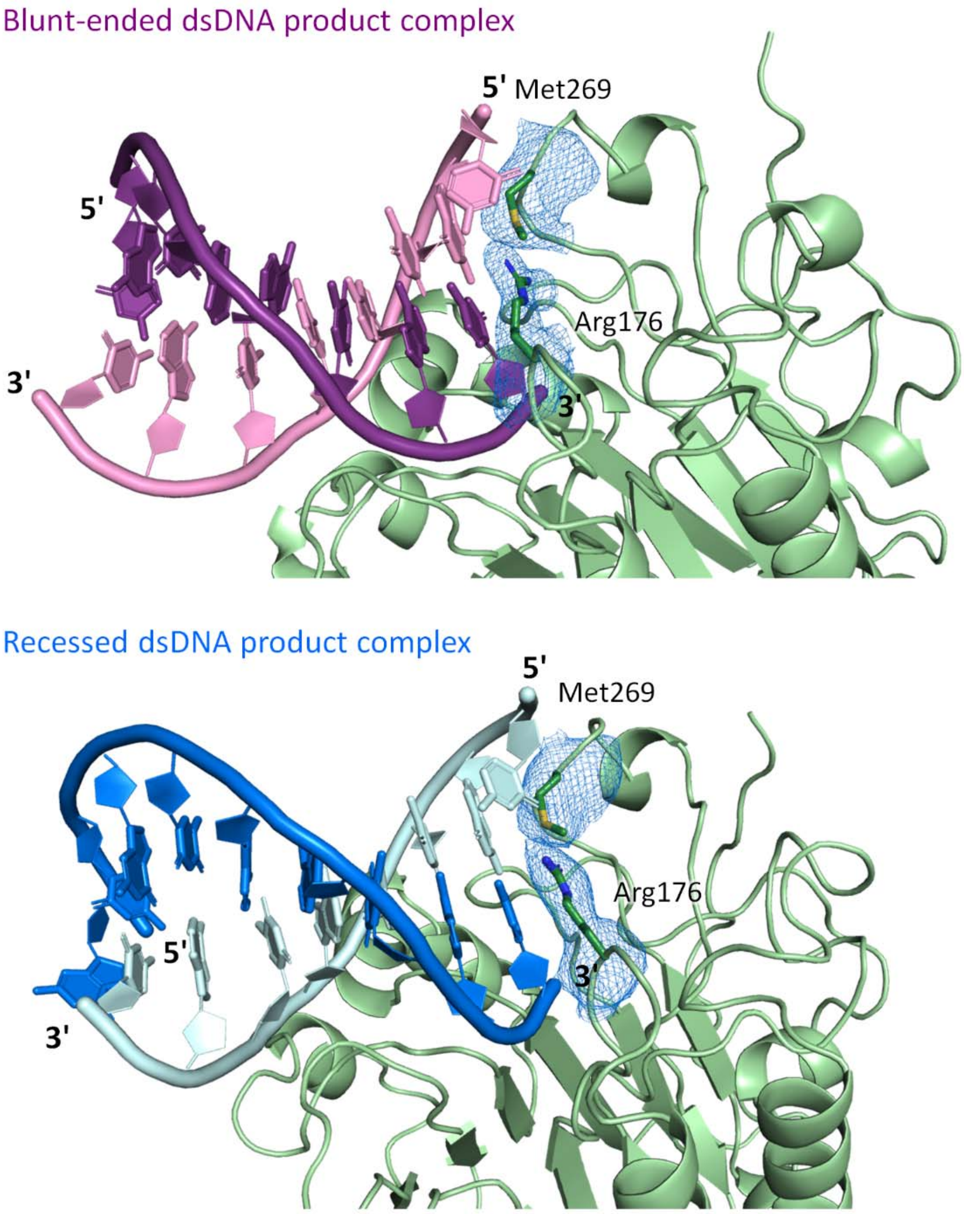

Supplementary Fig. 7: The omitted electron density maps of Arg176 and Met269 in mAPE1-dsDNA product complexes. The omitted electron density maps (Fo − Fc, 2.0 σ) of Arg176 and Met269 in mAPE1-blunt-ended dsDNA complex and mAPE1-recessed dsDNA complex structures.

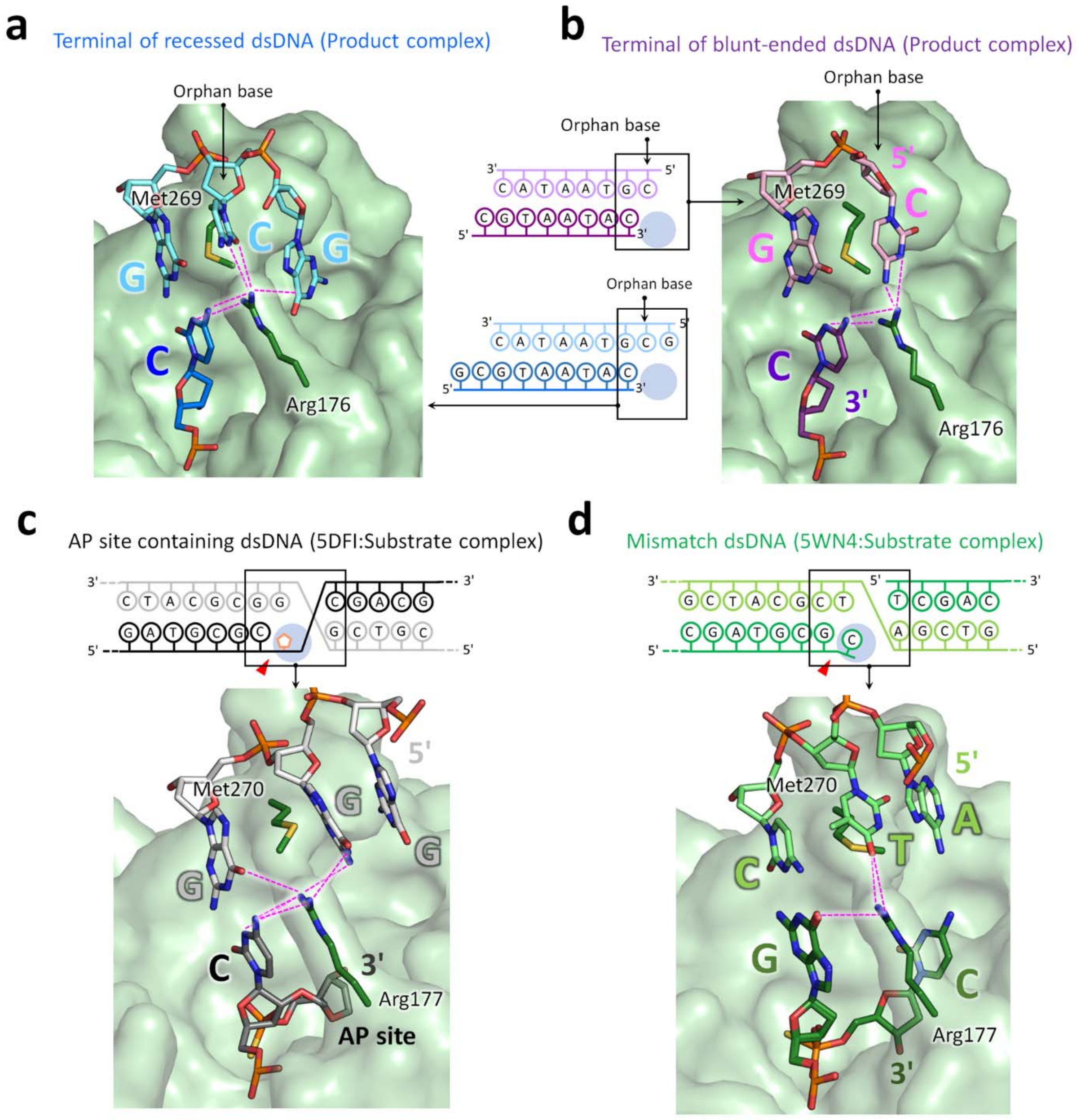

Supplementary Fig. 8: The interacting bases of RM bridge in various APE1-dsDNA complex structures. **a** Interacting bases in the mAPE1-recessed dsDNA product complex. **b** Interacting bases in the mAPE1-blunt-ended-dsDNA product complex. **c** Interacting bases in the hAPE1-AP site contained dsDNA complex (PDB entry: 5DFI). **d** Interacting bases in the hAPE1-mismatched dsDNA complex (PDB entry: 5WN4).

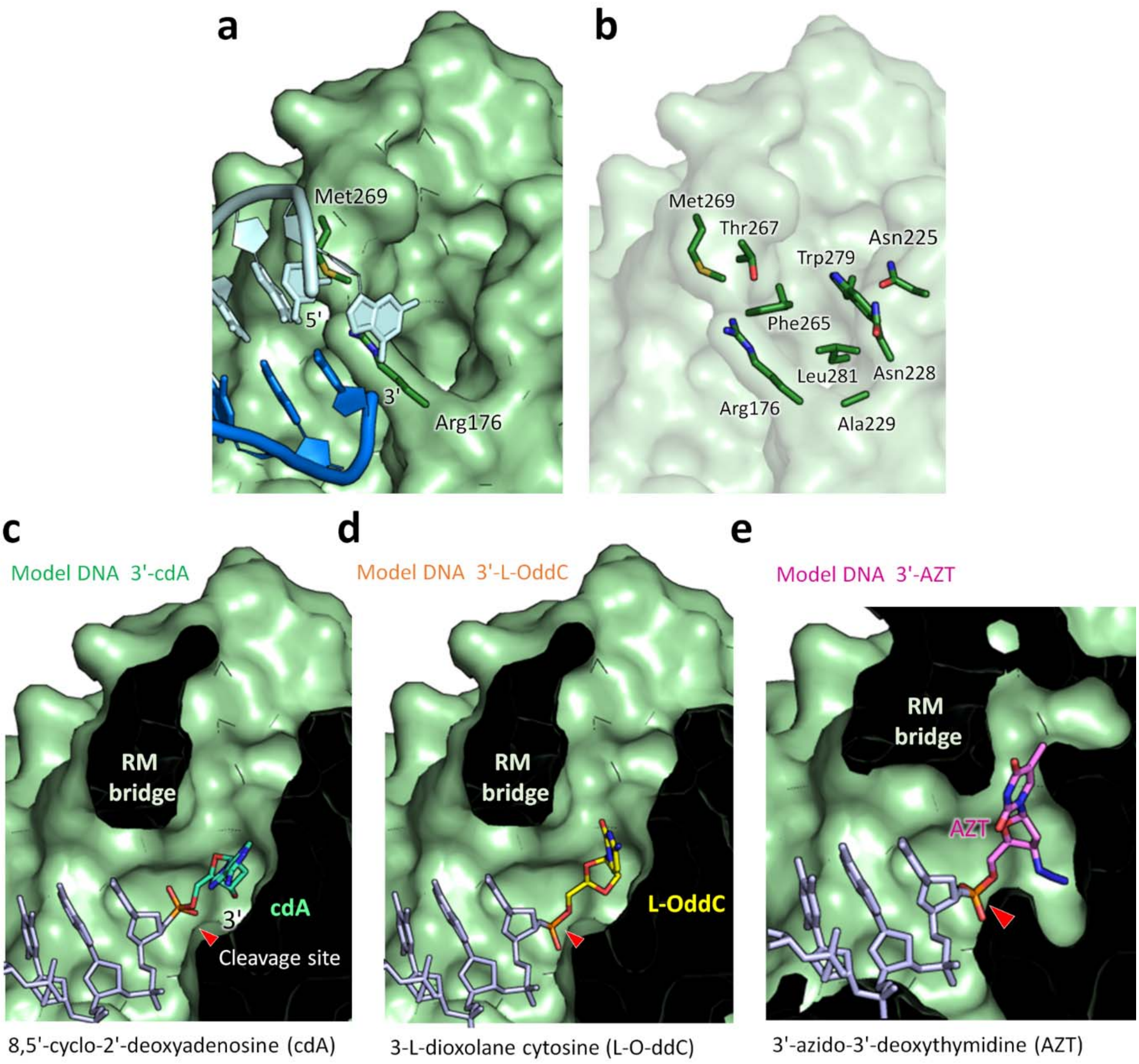

Supplementary Fig. 9: The structures of empty or nucleotide bound product pocket in the active site. a-b Surface structure and key residues in the product pocket. **c-e** The close-up view of various bases in the product pocket, including cdA, L-O-ddC and AZT. The damage bases were generated by molecular dynamics simulation. The red triangles highlight the position of the cutting site in the active site of APE1.

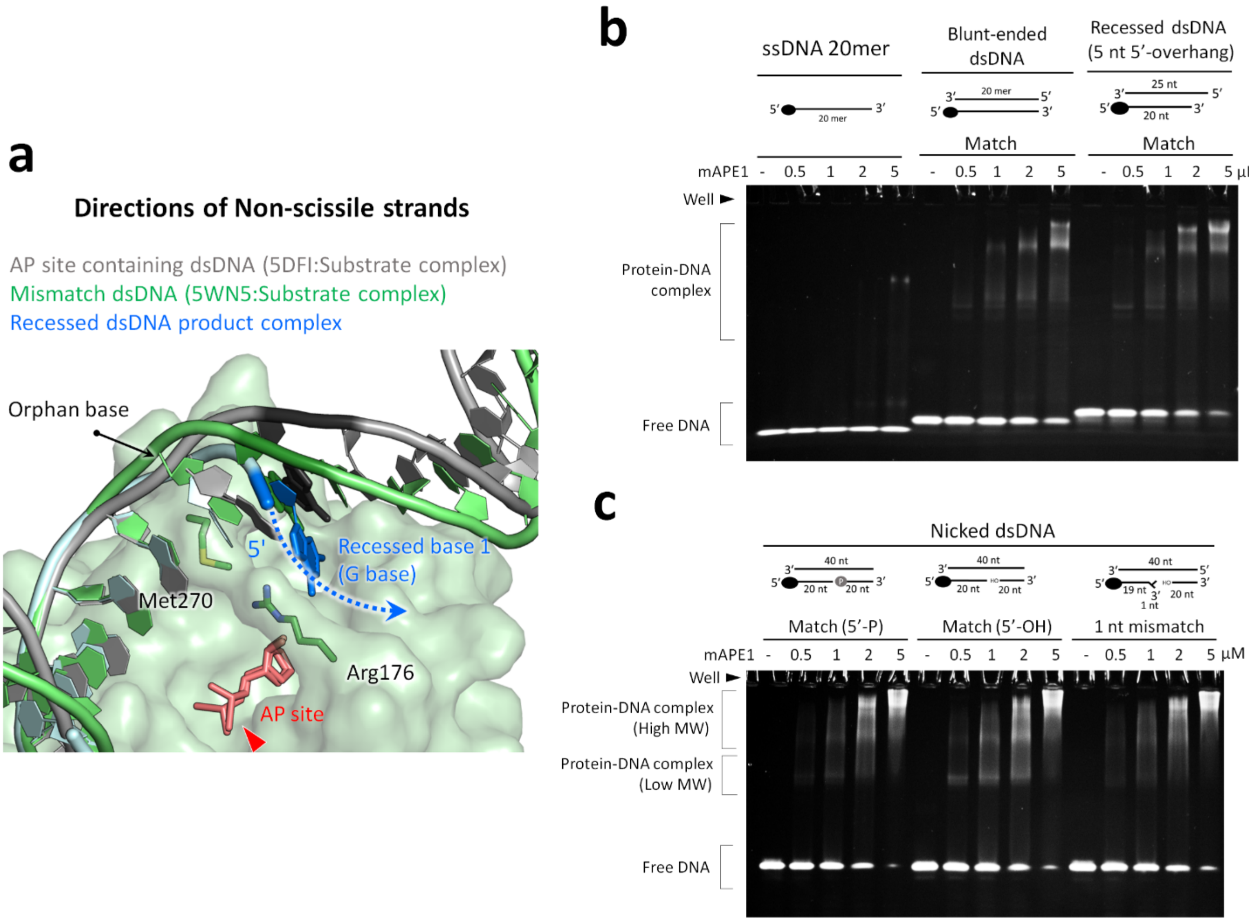

Supplementary Fig. 10: Structural analysis and Electrophoretic mobility shift assay (EMSA) measurements of APE1 in complex with various dsDNA. a Superposition of non-scissile strands of the three types of dsDNA indicates the extended area of the 5′-overhang in mAPE1-recessed-dsDNA product complex did not fit well to that on other dsDNAs, such as matched dsDNA and AP site-containing dsDNA. The extension area of recessed dsDNA was represented by a blue dotted arrow. **b** Binding assays of full-length mAPE1 on different DNA substrates, including ssDNA, blunt-ended dsDNA and recessed dsDNA with 5 nt-long 5′-overhang. The band of protein-DNA complex of blunt-ended dsDNA and recessed dsDNA can be observed at the mAPE1 concentration of 1 μM. **c** The substrates for EMSA measurements were nicked dsDNA with phosphoryl or hydroxyl groups at 5′ margin and 1 nt mismatched nicked dsDNA. The nicked dsDNA with phosphoryl at 5′ margin is with lower binding ability than the nicked dsDNA with 5′ hydroxyl groups. **b-c** Source data are provided as a Source Data file.

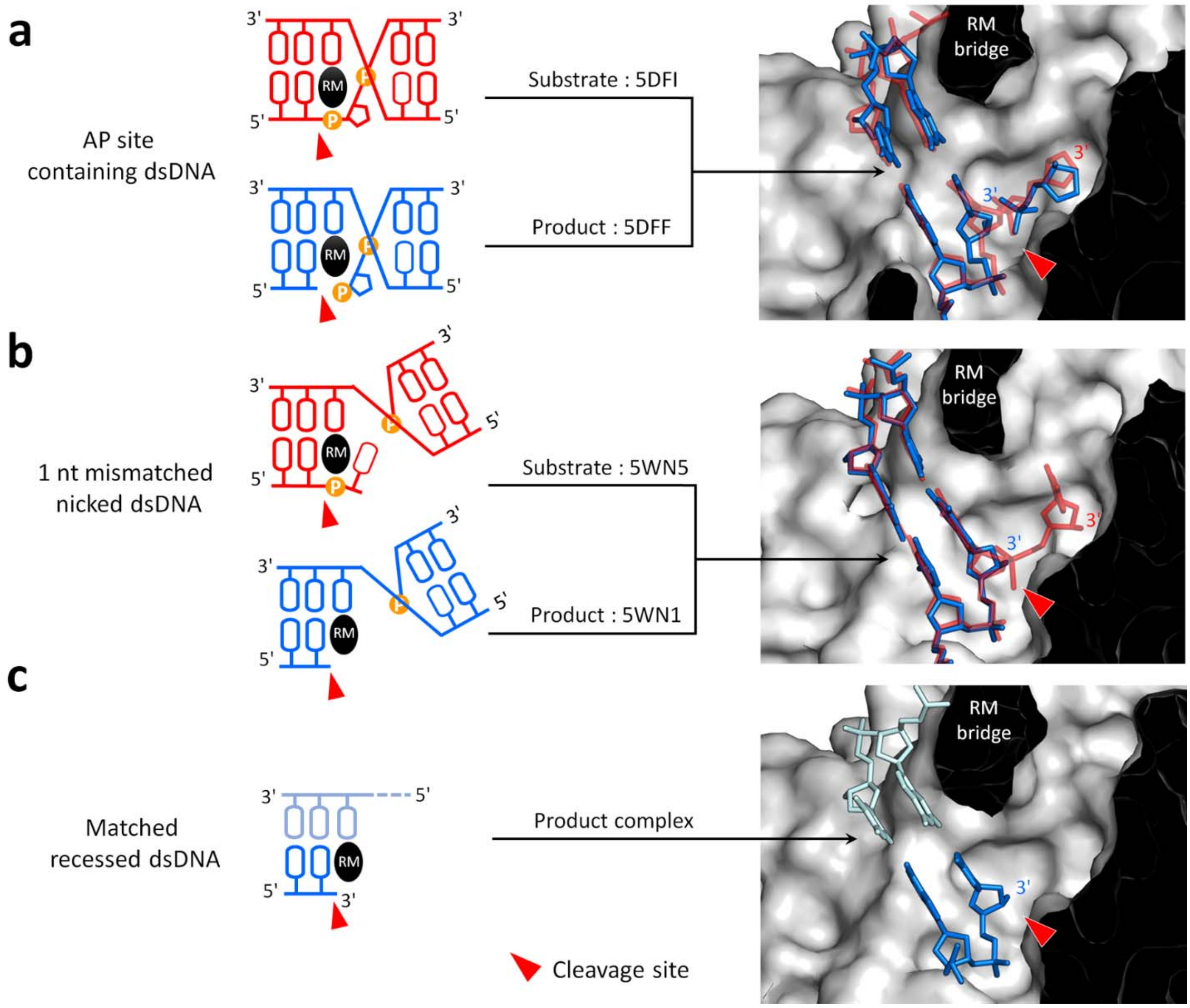

Supplementary Fig. 11: Structural comparison of APE1- dsDNA substrate complexes and APE1-dsDNA product complexes in endo- and exonuclease manner. Structural alignment of APE1-substrate and APE1-product complexes, including 3 different working modes, such as APE1 processes AP site-containing dsDNA in an endonuclease manner **a**, APE1 processes 1 nt mismatched nicked dsDNA in an exonuclease manner **b** and APE1 processes matched recessed dsDNA in an exonuclease manner **c**. Substrate dsDNAs are colored in red and product dsDNAs are colored in blue. The results show the cleavage sites are consistent in the 3 different working modes.

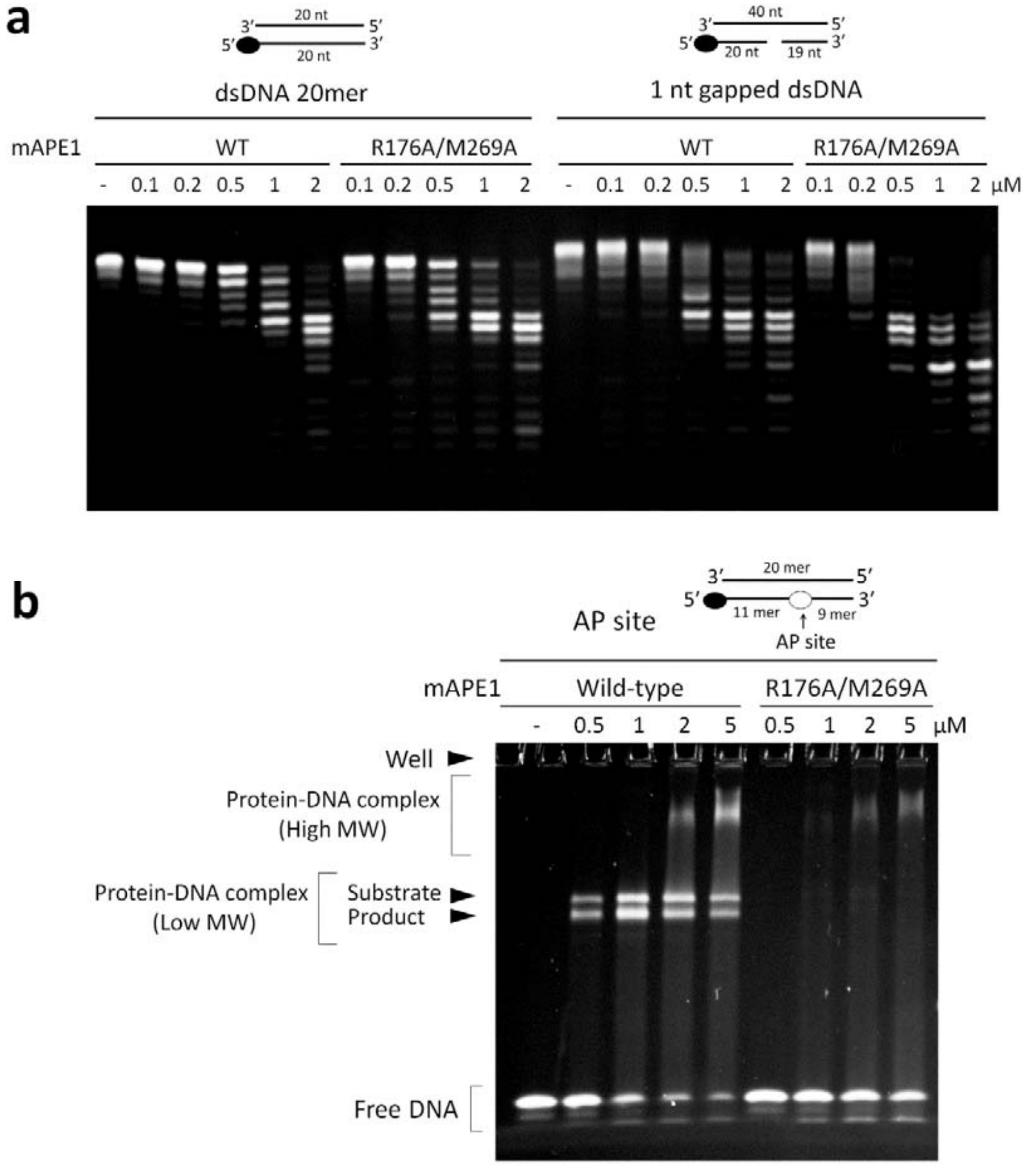

Supplementary Fig. 12: Exonuclease activity assay and binding assay of wild-type and double mutant (R176A/M269A) mAPE1. a The exonuclease activity assays for wild-type and double mutant (R176A/M269A) of mAPE1. When the substrates are blunt-ended dsDNA 20 mer or matched 1-nt-gapped dsDNA, the exonuclease activity of double mutant is higher than wild-type mAPE1. **b** EMSA measurements of wild-type and double mutant (R176A/M269A) mAPE1 binding to AP site-containing dsDNA. The band of protein-DNA complex with a lower molecular weight is not observed in the binding of mAPE1 double mutant. **a-b** Source data are provided as a Source Data file.
