# Supplementary material for: APE1 distinguishes DNA substrates in exonucleolytic cleavage by induced space-filling
Source: Nat Commun. 2021 Jan 27;12:601. doi: 10.1038/s41467-020-20853-2 (PMC7841161; doi:10.1038/s41467-020-20853-2)
Supplement: Supplementary file 3 — Reporting Summary [file 41467_2020_20853_MOESM3_ESM.pdf]

nature research

Corresponding author(s): Yu-Yuan Hsiao
Last updated by author(s): Dec 8, 2020

Reporting Summary

Nature Research wishes to improve the reproducibility of the work that we publish. This form provides structure for consistency and transparency in reporting. For further information on Nature Research policies, see our Editorial Policies and the Editorial Policy Checklist.

Statistics

For all statistical analyses, confirm that the following items are present in the figure legend, table legend, main text, or Methods section.

n/a	Confirmed	
☐	☒	The exact sample size (n) for each experimental group/condition, given as a discrete number and unit of measurement
☐	☒	A statement on whether measurements were taken from distinct samples or whether the same sample was measured repeatedly
☒	☐	The statistical test(s) used AND whether they are one- or two-sided<br>*Only common tests should be described solely by name; describe more complex techniques in the Methods section.*
☐	☒	A description of all covariates tested
☐	☒	A description of any assumptions or corrections, such as tests of normality and adjustment for multiple comparisons
☒	☐	A full description of the statistical parameters including central tendency (e.g. means) or other basic estimates (e.g. regression coefficient) AND variation (e.g. standard deviation) or associated estimates of uncertainty (e.g. confidence intervals)
☒	☐	For null hypothesis testing, the test statistic (e.g. F, t, r) with confidence intervals, effect sizes, degrees of freedom and P value noted<br>*Give P values as exact values whenever suitable.*
☒	☐	For Bayesian analysis, information on the choice of priors and Markov chain Monte Carlo settings
☐	☒	For hierarchical and complex designs, identification of the appropriate level for tests and full reporting of outcomes
☒	☐	Estimates of effect sizes (e.g. Cohen's d, Pearson's r), indicating how they were calculated

Our web collection on statistics for biologists contains articles on many of the points above.

Software and code

Policy information about availability of computer code

Data collection	X-ray diffraction data were collected at National Synchrotron Radiation Research Center, beamlines TLS 13B1, TLS 13C1, and TPS 05A in Taiwan. Data were indexed, integrated, and scaled using the program HKL2000.
Data analysis	Data were refined using the program Phenix version 1.9-1692, the model built by using Wincoot 0.8.9, and graphics were generated using PyMOL(TM) 2.0.7. The structural models were generated by CHARMM, CHARMM36, and CGenFF.

For manuscripts utilizing custom algorithms or software that are central to the research but not yet described in published literature, software must be made available to editors and reviewers. We strongly encourage code deposition in a community repository (e.g. GitHub). See the Nature Research guidelines for submitting code & software for further information.

Data

Policy information about availability of data

All manuscripts must include a data availability statement. This statement should provide the following information, where applicable:

- Accession codes, unique identifiers, or web links for publicly available datasets
- A list of figures that have associated raw data
- A description of any restrictions on data availability

The datasets generated and/or analyzed in the current study are available from the corresponding author on reasonable request. The coordinates of all the structures addressed in this work are available in the Protein Data Bank (wwPDB), including the structures determined in this study (PDB IDs 7CD5 and 7CD6) and in previous studies (PDB IDs 1HD7, 5DFI, 5DFF, 5WN0, 5WN1, 5WN4 and 5WN5). The source data of all the biochemical assays in this study are provided as a Source Data file.

Field-specific reporting

Please select the one below that is the best fit for your research. If you are not sure, read the appropriate sections before making your selection.

☒ Life sciences ☐ Behavioural & social sciences ☐ Ecological, evolutionary & environmental sciences

For a reference copy of the document with all sections, see nature.com/documents/nr-reporting-summary-flat.pdf

Life sciences study design

All studies must disclose on these points even when the disclosure is negative.

Sample size	For structure determination, over 10 different X-ray diffraction data sets were collected. The structure determination utilized the data set with the highest resolution, the best R-merge, and the fullest completeness. For biochemical assays, the gel base experiments were repeated multiple times according to standard protocols.
Data exclusions	Following the convention of X-ray diffraction data collection, the over-saturated spots are excluded by HKL2000 based on the general criteria.
Replication	The nuclease activity assays and nucleic acid binding assays in this study are gel based. All measurements have been repeated at least three times to ensure reproducibility.
Randomization	For the X-ray structure refinement, the R-free set is randomly selected by the CCP4 program. Randomization is not relevant for the nuclease activity assays and nucleic acid binding assays.
Blinding	In this work, measurable structures and biochemical activities are utilized to understand APE1 functionality, and blinding is not relevant.

Reporting for specific materials, systems and methods

We require information from authors about some types of materials, experimental systems and methods used in many studies. Here, indicate whether each material, system or method listed is relevant to your study. If you are not sure if a list item applies to your research, read the appropriate section before selecting a response.

Materials & experimental systems

n/a	Involved in the study
☒	☐ Antibodies
☒	☐ Eukaryotic cell lines
☒	☐ Palaeontology and archaeology
☒	☐ Animals and other organisms
☒	☐ Human research participants
☒	☐ Clinical data
☒	☐ Dual use research of concern

Methods

n/a	Involved in the study
☒	☐ ChIP-seq
☒	☐ Flow cytometry
☒	☐ MRI-based neuroimaging
